# Isoeugenol Inhibits Adipogenesis in 3T3-L1 Preadipocytes with Impaired Mitotic Clonal Expansion

**DOI:** 10.3390/nu16091262

**Published:** 2024-04-24

**Authors:** Yae Rim Choi, Hyun-Jin Na, Jaekwang Lee, Young-Suk Kim, Min Jung Kim

**Affiliations:** 1 Division of Food Functionality Research, Korea Food Research Institute, Wanju-gun 55365, Republic of Korea; uiu7896@gmail.com (Y.R.C.); hjna@kfri.re.kr (H.-J.N.); jklee@kfri.re.kr (J.L.); 2 Department of Food Science and Biotechnology, Ewha Womans University, Seoul 03760, Republic of Korea; yskim10@ewha.ac.kr

**Keywords:** isoeugenol, anti-adipogenesis, mitotic clonal expansion (MCE), cell cycle arrest, reactive oxygen species (ROS)

## Abstract

Isoeugenol (IEG), a natural component of clove oil, possesses antioxidant, anti-inflammatory, and antibacterial properties. However, the effects of IEG on adipogenesis have not yet been elucidated. Here, we showed that IEG blocks adipogenesis in 3T3-L1 cells at an early stage. IEG inhibits lipid accumulation in adipocytes in a concentration-dependent manner and reduces the expression of mature adipocyte-related factors including PPARγ, C/EBPα, and FABP4. IEG treatment at different stages of adipogenesis showed that IEG inhibited adipocyte differentiation by suppressing the early stage, as confirmed by lipid accumulation and adipocyte-related biomarkers. The early stage stimulates growth-arrested preadipocytes to enter mitotic clonal expansion (MCE) and initiates their differentiation into adipocytes by regulating cell cycle-related factors. IEG arrested 3T3-L1 preadipocytes in the G_0_/G_1_ phase of the cell cycle and attenuated cell cycle-related factors including cyclinD1, CDK6, CDK2, and cyclinB1 during the MCE stage. Furthermore, IEG suppresses reactive oxygen species (ROS) production during MCE and inhibits ROS-related antioxidant enzymes, including superoxide dismutase1 (SOD1) and catalase. The expression of cell proliferation-related biomarkers, including pAKT and pERK1/2, was attenuated by the IEG treatment of 3T3-L1 preadipocytes. These findings suggest that it is a potential therapeutic agent for the treatment of obesity.

## 1. Introduction

Obesity arises from an imbalance between energy intake and expenditure [1] and leads to metabolic diseases, including cardiovascular diseases, diabetes, and liver diseases [2,3]. Adipogenesis plays a crucial role in obesity; it is the process by which preadipocytes differentiate into mature adipocytes, which accumulate triglycerides and maintain energy homeostasis [4]. The adipogenesis of 3T3-L1 preadipocytes begins with differentiation by hormonal stimulation (MDI cocktail: 3-isobutyl-1-methylxanthine (IBMX), dexamethasone, and insulin) in a state of arrested growth [5]. Growth-arrested 3T3-L1 cells then re-enter the cell cycle and undergo up to two cycles of mitosis before undergoing mitotic clonal expansion (MCE) during the early stages of differentiation [6]. This is followed by terminal differentiation, in which mature adipocytes develop [7]. As the initiation of adipogenesis involves entry into the MCE, MCE can serve as an attractive target for anti-adipogenic strategies.

The early stage, which includes MCE, is regulated by hormones, growth factors, and transcription factors [8]. IBMX and insulin increase cAMP levels, which in turn enhance PKA activity, cAMP response element binding protein (CREB) activity, and CCAAT/enhancer binding protein β (C/EBPβ) expression [9]. Dexamethasone activates C/EBPβ, which activates transcription by binding at specific elements within the proximal promoter regions of both the peroxisome proliferator-activated receptor γ (PPARγ) and C/EBPα genes, which are crucial for the ultimate differentiation of adipocytes [10]. In addition, insulin stimulates pathways such as AKT that promote cell cycle progression and differentiation [11]. This involves a process by which cells prepare to grow and divide, particularly by promoting the transition from the G_1_ to the S phase of the cell cycle. The MCE stage also involves the activation of signaling pathways such as extracellular signal-regulated protein kinase (ERK) and the production of reactive oxygen species (ROS), which can further influence the differentiation process by regulating transcription factors such as PPARγ and additional C/EBP family members [12,13]. Therefore, if the early stage is affected by a specific substance, various related factors may change.

Isoeugenol (IEG) is known as propenyl-substitute guaiacol, which has a structure in which the double bond of the alkyl side chain is shifted by one carbon. IEG is found in various plants, including cloves, calamus, and nutmeg [14,15]. IEG can be obtained by directly extracting the essential oil of clove trees and is used as a precursor for the synthesis of natural vanillin, a food additive [16]. IEG has biologically promising antioxidant, anti-inflammatory, and anti-bacterial properties [17,18]. Additionally, clove, which contains IEG, can reduce body weight and abdominal adipose tissue in high-fat diet (HFD)-induced mouse in 1% alcohol extract [19]. There have been no specific studies on the direct effects of IEG on obesity or adipogenesis. However, the structural isomer of IEG, eugenol, is the primary aromatic compound in clove oil and is associated with the attenuation of weight accumulation and changes in the composition and function of the gut microbiota in a mouse model induced by an HFD [20]. Further research is needed to learn more about IEG.

In the present study, we investigated the effects of IEG on adipogenesis in 3T3-L1 cells and its underlying molecular mechanisms. We focused on the early stages of lipid differentiation.

## 2. Materials and Methods

### 2.1. Materials

3T3-L1 mouse embryonic fibroblast cell was purchased from the American Type Culture Collection (ATCC; Manassas, VA, USA). Dulbecco’s modified Eagle’s medium (DMEM), newborn calf serum (BCS), penicillin/streptomycin (P/S), and fetal bovine serum (FBS) were obtained from Gibco Inc. (Grand Island, NY, USA). Isoeugenol, insulin, dexamethasone, 3-isobuty-1-methylxanthine (IBMX), isopropanol, formaldehyde, Oil Red O (ORO), RIPA buffer, bovine serum albumin (BSA), and propidium iodide (PI) were obtained from Sigma-Aldrich (St. Louis, MO, USA).

### 2.2. Cell Culture and Adipocyte Differentiation

3T3-L1 preadipocytes were grown in DMEM containing 10% BCS and 1% P/S at 37 °C with 5% CO_2_. 3T3-L1 preadipocytes were differentiated by differentiation medium (MDI; DMEM containing 10% FBS, 1% P/S, IBMX (0.5 mM), dexamethasone (1 μM), and insulin (1 μg/mL). After incubation for 48 h, the medium was replaced every 2 days with DMEM containing 10% FBS and insulin (1 μg/mL) until day 8.

### 2.3. Oil Red O (ORO) Staining

3T3-L1 cells were fixed with 4% formaldehyde for 10 min and washed twice with PBS. Fixed cells were washed with 60% isopropanol. The cells were stained with ORO working solution for 10 min and washed with distilled water. The lipid droplets were observed by bright field microscopy and dye in the cells was extracted with 100% isopropanol and quantified at OD_500_ using a microplate reader. The absorbance of IEG-treated cells (A_IEG_), blanks (A_blank_), and differentiated cells (A_control_) were calculated as (A_IEG_ − A_blank_)/A_blank_ × 100%

### 2.4. Western Blotting Analysis

For protein extraction, 3T3-L1 cells were lysed in RIPA buffer containing protease and phosphatase inhibitors and then centrifuged. All processes were carried out in ice conditions. The protein concentration of extracts was measured using Pierce^TM^ BCA protein assay (Thermo Fisher Scientific; Boston, MA, USA). Proteins were separated via sodium dodecyl sulfate-polyacrylamide gel electrophoresis (SDS-PAGE) and then transferred onto polyvinylidene difluoride (PVDF) membranes (Bio-Rad, Herculues, CA, USA). PVDF membranes were blocked with 5% BSA and incubated overnight with primary antibodies (1:1000) against PPARγ, C/EBPα, FABP4, C/EBPβ, cyclin D1, cyclin B1, cyclin-dependent kinase 6 (CDK6), CDK2, Akt, p-Akt, SOD1, catalase, phosphor-AKT, AKT, phosphor-ERK1/2, ERK1/2, phosphor-p38 mitogen-activated protein kinase (MAPK), p38 MAPK, and β-actin (Cell Signaling Technology; Danvers, MA, USA). After washing in TBS with Tween 20, the membranes were incubated with secondary antibodies (1:2000 dilution). The band signals were detected using enhanced chemiluminescence (ECL; Thermo Fisher Scientific) Western blotting detection kit. The reference protein is β-actin, which has no difference in expression levels between preadipocytes and adipocytes (Figure S1).

### 2.5. Quantitative Polymerase Chain Reaction (qPCR)

For RNA extraction, TRIzol reagent (Thermo Fisher Scientific) was used according to the manufacturer’s protocol. Total RNA (1 μg) was transformed into cDNA using a SuperScript III RT kit (Invitrogen, Carlsbad, CA, USA). cDNAs were amplified using the FastStart SYBR Green Master Mix (Applied Biosystems, Forster city, CA, USA) along with the following gene-specific primers: *Cebpa*, *Pparg*, *Fabp4*, *Adipoq*, *Fasn*, *Cebpb*, *cyclinD1*, *cdk6*, *cdk2*, and *18S*. The primer sequences are listed in Table 1. qPCR was performed using the QuantStudio 6 Flex Real-Time PCR System (Applied Biosystems). The reference gene with no significant difference in expression levels between preadipocytes and adipocytes was selected as *18S* (Figures S2 and S3). Data are expressed as fold differences normalized to *18S* levels.

### 2.6. Cell Proliferation

3T3-L1 preadipocytes were seeded in 96-well plates at a density of 1 × 10^4^ cells/well. Then, IEG was treated with various concentrations (0–100 μM). After 24 and 48 h, cells were treated with the Cell Counting Kit-8 (CCK-8; Dojindo, Kumamoto, Japan) and incubated for 2 h. Cell proliferation was determined by measuring the absorbance at 450 nm (A_450_) and reference absorbance at 650 nm (A_650_) using a microplate reader. Cell proliferation (%) was calculated by (A_450_ − A_600_)/A_control_ × 100%. The control (A_con_) is a 0 μM concentration of IEG.

### 2.7. Flow Cytometry

3T3-L1 cells were fixed with 70% ethanol at −20 °C overnight, centrifuged, and washed twice with PBS. Fixed cells were stained with PI (25 μg/mL) and RNase A (50 μg/mL). The cell cycle was analyzed using a CytoFLEX 3 Laser (Beckman Coulter, Brea, CA, USA). Data analysis was measured using CytExpert 2.4 software (Beckman Coulter).

### 2.8. Measurement of Intracellular ROS

3T3-L1 cells were stained with 5 μM 2′,7′-dichlorodihydrofluorescein diacetate (H_2_DCFDA, Thermo Fisher Scientific) for 30 min at 37 °C and washed with PBS. Intracellular ROS levels were monitored using a fluorescence microscope and quantified using a spectrophotometer (excitation wavelength: 495 nm and emission wavelength: 525 nm).

### 2.9. Statistical Analysis

The statistical significance of differences between the values of various experimental and control groups was determined by one-way analysis of variance (ANOVA) using Tukey’s post-hoc test. Data are expressed as the mean ± standard error (n ≥ 3). A value of statistical significance was considered at *p* < 0.05 significant. All analyses were determined using the GraphPad Prism 10 software (GraphPad Software, San Diego, CA, USA).

## 3. Results

### 3.1. IEG Inhibits MDI-Induced Adipogenesis in 3T3-L1 Cells

**Figure 1:**
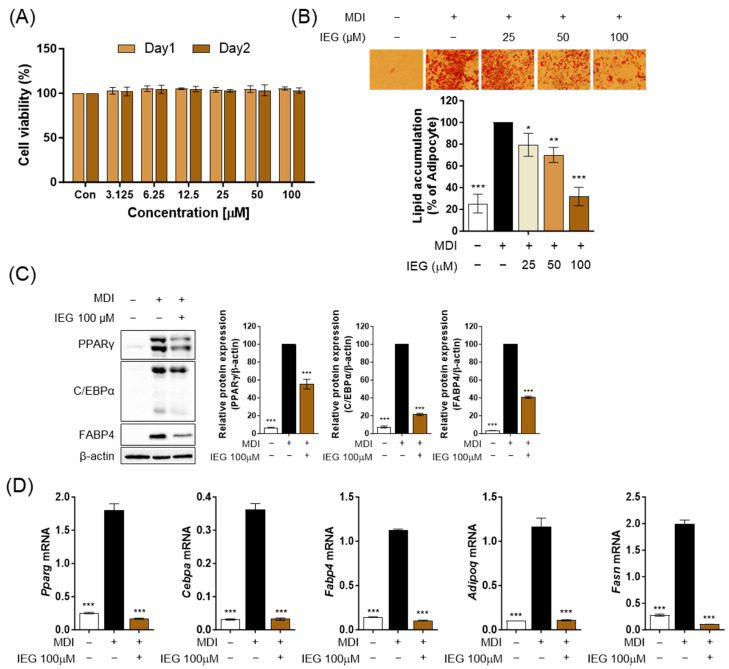
Effect of isoeugenol (IEG) on lipid accumulation and adipogenesis in 3T3-L1 adipocytes. (**A**) Viability of 3T3-L1 cells treated with IEG. (**B**) Oil red O (ORO) images and quantitative values of lipid accumulation in 3T3-L1 cells treated with IEG for 8 days in a dose-dependent manner. (**C**) Protein expression levels of PPARγ, C/EBPα, and FABP4 in 3T3-L1 adipocytes treated by IEG (100 μM) at day 8. β-Actin was used as a control. (**D**) Quantification of mRNA levels of *Pparg*, *Cebpa*, *Fabp4*, *Adipoq*, and *Fasn* in 3T3-L1 adipocytes treated by IEG (100 μM) at day 8. Data are expressed as the mean ± SD (n = 3). * *p* < 0.05, ** *p* < 0.01, and *** *p* < 0.001 when compared to the adipocytes.

Firstly, we observed the cell viability after treating the cells with various concentrations of IEG (3.125–100 μM) to determine the treatment concentration of IEG. No change in cell viability was observed at any concentration of IEG after 24 and 48 h (Figure 1A). Therefore, we selected three high concentrations (25, 50, and 100 μM) and examined the effects of IEG during the differentiation process of 3T3-L1 cells. According to ORO images and quantified values, MDI effectively induced 3T3-L1 differentiation into adipocytes, resulting in a 75% increase in lipid accumulation compared to 3T3-L1 preadipocytes (Figure 1B). In contrast, IEG decreased the accumulation of lipid droplets in a dose-dependent manner, with reductions quantified at 79, 70, and 32% for doses of 25, 50, and 100 μM, respectively. The protein expression levels of mature adipocyte transcription factors including PPARγ, C/EBPα, and FABP4 were significantly reduced by IEG at 100 μM, which had the highest inhibitory efficacy in lipid accumulation (Figure 1C). Furthermore, the mRNA expression levels of adipogenesis-related genes, including *Pparg*, *Cebpa*, *Fabp4*, and *Adipoq,* as well as the lipogenic gene *Fasn* were suppressed by IEG (Figure 1D). Thus, IEG suppressed adipogenesis in 3T3-L1 cells.

### 3.2. IEG Suppresses the Early Stage of Adipocyte Differentiation

**Figure 2:**
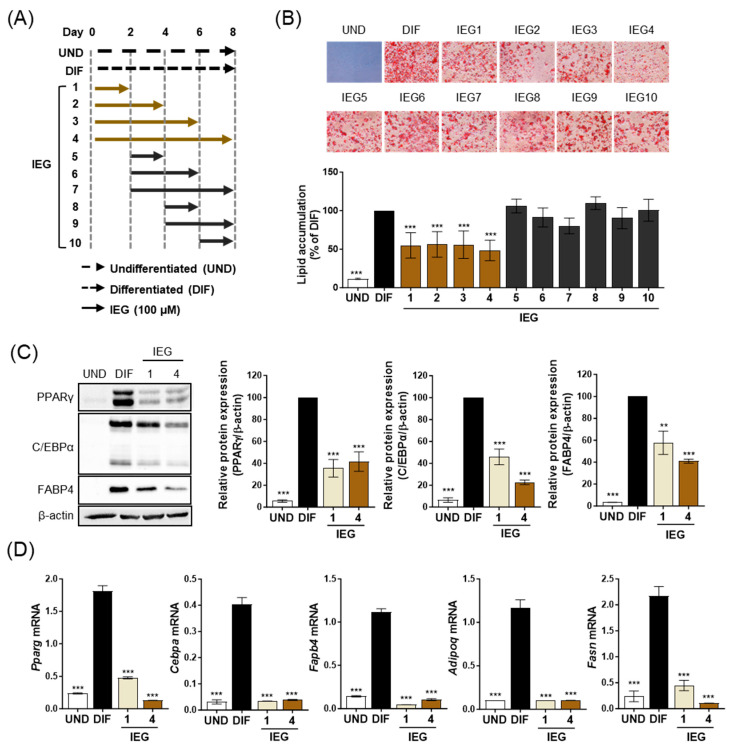
IEG influence on the early stage of adipogenesis in 3T3-L1 adipocytes. (**A**) Schematic diagram of IEG treatment for 8 days during the differentiation process. IEG (100 μM) was treated in 3T3-L1 cells at the indicated times. (**B**) ORO images and quantitative values of lipid accumulation in 3T3-L1 cells treated with 100 μM IEG at day 8. (**C**) Protein expression levels of PPARγ, C/EBPα, and FABP4 in IEG-treated 3T3-L1 cells. (**D**) Quantification of mRNA levels of *Pparg*, *Cebpa*, *Fapb4*, *Adipoq*, and *Fasn* at day 8. Data are expressed as the mean ± SD (n = 3). ** *p* < 0.01 and *** *p* < 0.001 when compared to the adipocytes. UND: preadipocytes, DIF: adipocytes.

To elucidate the inhibitory mechanism of IEG on adipogenesis, the effect of IEG on adipogenesis was observed at different time points (Figure 2A). Among 10 different IEG treatment periods, IEG (100 μM) significantly inhibited MDI-induced lipid accumulation only in the IEG1 to IEG4 and not in the IEG5 to IEG10. For IEG1 and IEG4, the IEG was treated for the first 2 days (days 0–2) and, in particular, the initial 2-day treatment (IEG1) achieved the same adipogenesis inhibition efficacy as that of the 8-day treatment (IEG4). Comparing only IEG1 and IEG4, the protein expression levels of terminal adipocyte markers (PPARγ, C/EBPα, and FABP4), which were increased in DIF, were significantly decreased in IEG1 and IEG4 (Figure 2C). The expression levels of adipogenesis-related genes, including *Pparg*, *Cebpa*, *Fabp4*, *Adipoq*, and *Fasn*, were also significantly decreased in IEG1 and IEG4 compared with those in DIF (Figure 2D). These results suggest that the critical period for the suppression of 3T3-L1 adipogenesis by IEG is the first 2 days.

### 3.3. IEG Suppresses MDI-Induced Cell Cycle Progression in the Early Stage of Differentiation

**Figure 3:**
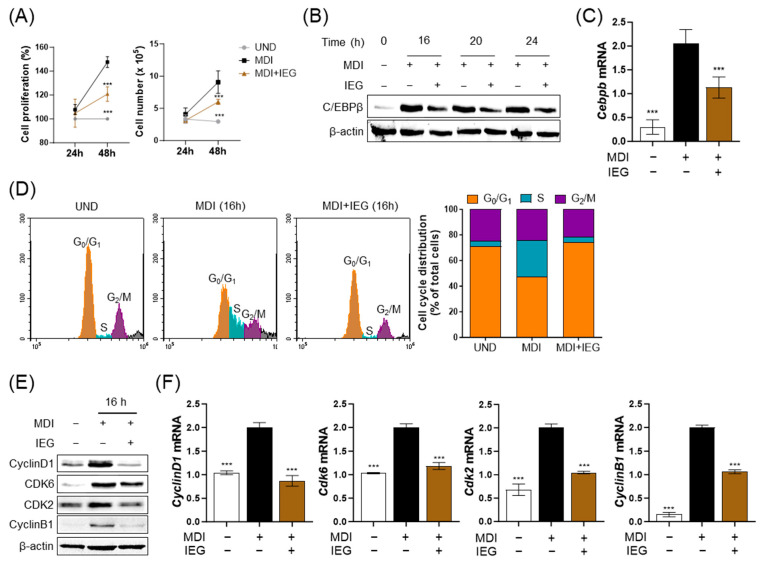
Effect of IEG on mitotic clonal expansion (MCE) process of adipogenesis. (**A**) IEG-induced changes in 3T3-L1 cell proliferation for 48 h. (**B**) Time-course analysis of protein expression level of C/EBPβ following 24 h treatment with IEG. (**C**) The mRNA expression level of *Cebpb* in 16 h IEG treatment. (**D**) Flow cytometry of cell cycle progression of IEG-treated cells. Gray area: debreis and aggregates. (**E**,**F**) Changes in protein (**E**) and mRNA (**F**) expression levels of cyclin D1, Cdk6, Cdk2, and cyclin B1. *** *p* < 0.001 when compared to the adipocytes. UND: preadipocytes, DIF: adipocytes.

During the differentiation process of 3T3-L1 cells, the first 2 days corresponded to the early stage, especially the MCE period. Therefore, we examined the effect of IEG on MCE. The proliferation and number of MDI-induced 3T3-L1 cells increased from 24 to 48 h (Figure 3A). However, MDI-induced cells with IEG significantly decreased proliferation and cell numbers. Observation of C/EBPβ expression during the first 24 h showed that IEG suppressed C/EBPβ expression which was elevated by MDI starting at 16 h after treatment (Figure 3B,C). The effects of IEG on MDI-induced cell cycle progression were analyzed using flow cytometry (Figure 3D). The cell cycle of undifferentiated cells (UND) was arrested at the G_0_/G_1_ phase, whereas the cells in the MDI group entered the S phase at 16 h. The cell populations of UND and DIF in G_0_/G_1_ phase were 71.21% and 47.42%, whereas those in the S phase were 4.08% and 28.58%, respectively. In the case of the IEG-treated group, the cell populations in the G_0_/G_1_ and S phases were 74.44% and 3.90%, respectively. These data suggested that IEG inhibited the cell cycle transition from G_0_/G_1_ into the S phase. IEG treatment for 16 h also affected the expression of cell cycle-related factors (Figure 3E,F). IEG significantly decreased the expression of cyclin D1, CDK6, CDK2, and cyclin B1 in MDI-treated cells after 16 h. Collectively, these data suggested that IEG inhibited adipogenesis in the early stages of adipocyte proliferation and differentiation by regulating cell cycle progression.

### 3.4. IEG Reduces Intracellular ROS Generation in the Early Stage of Differentiation

**Figure 4:**
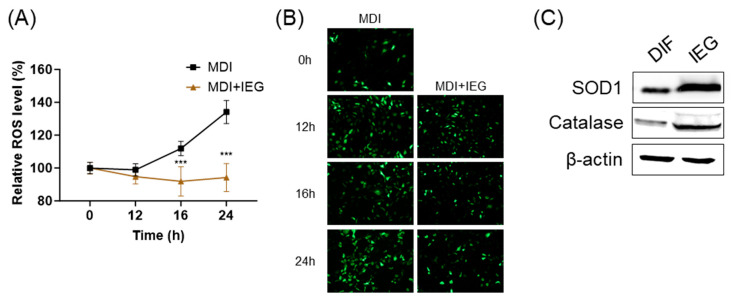
Inhibitory effect of intracellular reactive oxygen species (ROS) generation by IEG during the MCE in adipocyte differentiation. (**A**,**B**) Quantitative values (**A**) and representative fluorescence images (**B**) of changes in intracellular ROS levels over time after treatment with MDI and IEG (100 μM). (**C**) Protein expression of ROS antioxidant enzymes, SOD1, and catalase at day 8. *** *p* < 0.001 compared to the MDI-only-treated group at the same time point.

The effects of IEGs on intracellular ROS levels in the MCE stage were measured after 12, 16, and 24 h of treatment. In preadipocytes treated with MDI, ROS levels increased in a time-dependent manner; however, when treated with IEG (100 μM), ROS levels were significantly decreased at 16 and 24 h compared to the MDI group (Figure 4A,B). Moreover, on day 8 of adipogenic differentiation, antioxidant enzyme proteins, including SOD1 and catalase, increased compared to that in adipocytes (Figure 4C).

### 3.5. IEG Attenuated the Expression of AKT and ERK in the Early Stage of Differentiation

**Figure 5:**
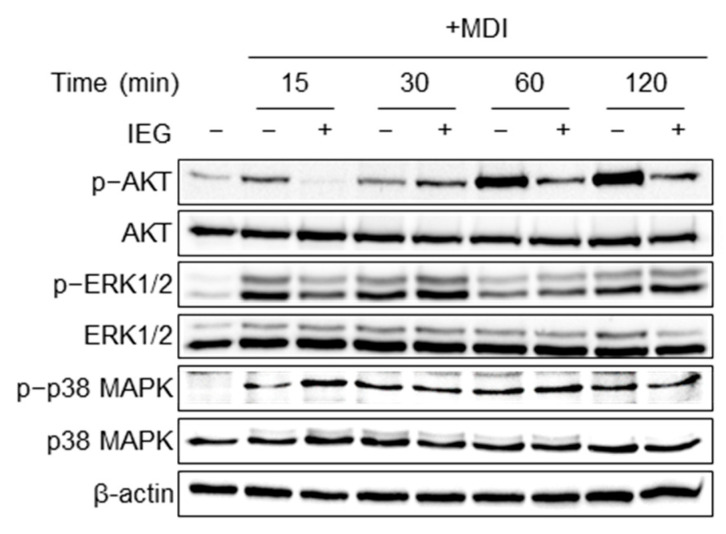
Effect of IEG on cell proliferation signaling pathways regulating MCE.

Previous studies have shown that AKT, ERK1/2, and p38 MAPK signaling pathways may be involved in adipogenesis [21]. IEG inhibits cell proliferation in the early stages of differentiation; therefore, we investigated the pathways involved. As shown in Figure 5, MDI treatment increased the expression of p-AKT and p-ERK1/2 at 15 min, with p-AKT reaching its maximum level at 60 min. In contrast, IEG treatment attenuated the expression of p-AKT and p-ERK1/2 after 15 min. The expression of p-p38 also increased; however, no changes were observed in response to IEG.

## 4. Discussion

In the present study, we investigated the anti-adipogenic effects of IEG and the underlying molecular mechanisms in 3T3-L1 cells. The treatment with IEG during 3T3-L1 adipocyte differentiation inhibited lipid accumulation in a dose-dependent manner and suppressed the protein and mRNA expressions of PPARγ, C/EBPα, and FABP4 on day 8. Although the anti-obesity effects of clove extract, which contains IEG, and eugenol, an isomer of IEG, have been studied, this study is the first to investigate these effects.

Adipogenesis progresses in multiple stages [7,22]. The results showed that IEG is involved in the early stage (days 0–2) and modulates the transcription factors (PPARγ, C/EBPα, and FABP4). The process of differentiating 3T3-L1 preadipocytes into mature adipocytes during the early stage, especially MCE, is essential for adipogenesis and involves PPARγ, C/EBPα, and FABP4 expression [5]. During the MCE period, committed preadipocytes undergo several rounds of cell division [6]. This expansion is not just a simple increase in the cell number; it is also a necessary step for cells to proceed with the differentiation process. The MCE phase is characterized by increased cell proliferation and is typically regulated by various growth factors, hormones, and transcription factors. Preadipocytes are arrested in the G_1_ phase of the cell cycle by CDK inhibitory proteins (p27^Kip1^ and p21^Waf1/Cip1^) and the hypophosphorylated tumor suppressor retinoblastoma protein. Under MDI treatment, the growth-arrested 3T3-L1 cells simultaneously express C/EBPβ and C/EBPδ and re-enter the cell cycle [6,23]. Cyclin D and CDK4/CDK6 complexes act as regulators of the early G_1_ phase [24], whereas cyclin E and CDK2 complexes are critical for the transition between the G_1_ and S phases [25]. Together, they help cells arrested in the cell cycle to re-enter the cycle, thereby enabling their progression to the G_1_/S phase transition. The entry of the S phase occurred approximately 14 h after treatment with the hormonal cocktail and the highest DNA synthesis was observed approximately 18 h after MDI. Therefore, blocking or delaying cell cycle progression by suppressing cell cycle regulators and upregulating CDK inhibitors during MCE may be an efficient way to inhibit adipogenesis. Methyl cinnamate, a natural flavor compound, activated CaMKK2-AMPK signaling on day 2, the early stage of adipogenic differentiation, and inhibited the adipogenic transcription factors SREBP-1, PPARγ, C/EBPα, and adiponectin [26]. In addition, cinnamyl alcohol, a flavor compound, inhibited the cell cycle and C/EBPβ in MCE, thereby inhibiting differentiation into adipocytes [27]. Likewise, in our study, IEG treatment significantly decreased cell division and cell number and delayed the progression of cells into the G_2_/M phase by 24 h in the MCE stage. Additionally, C/EBPβ and cell cycle-related factors were reduced by IEG after 16 h of treatment.

During the MCE phase, cells actively grow and divide, consuming large amounts of oxygen, which leads to ROS production [28]. ROS can damage cells and are associated with an excessive supply of energy substrates in metabolic diseases and obesity [29]. However, at appropriate levels, they serve as important regulators of cellular signaling and function, including cellular proliferation, differentiation, survival, and apoptosis [13,30]. Hormone cocktail treatment of 3T3-L1 preadipocytes accelerates preadipocyte differentiation by generating ROS during MCE [13]. During adipogenesis, cellular ROS levels depend on the balance between the pro-oxidant enzymes responsible for ROS production and the antioxidant enzymes involved in reducing ROS [31]. Pro-oxidant enzymes, such as xanthine oxidase and NADPH oxidase, generate ROS, whereas antioxidant enzymes, such as catalase, SOD, glutathione reductase, and glutathione peroxidase, eliminate ROS. Extensive research has been conducted on potential anti-obesity agents that regulate key enzymes related to antioxidant reactions and metabolic pathways [32]. Resveratrol, a natural compound found in grapes and red wine, reduces oxidative stress by enhancing the activity of antioxidant enzymes, inhibiting pro-oxidant enzymes, and increasing the cellular capacity to scavenge and neutralize ROS [33]. Carnosic acid attenuates ROS levels by regulating NADPH oxidase 4 via the NF-κB signaling pathway in the early stage of adipogenesis [34]. IEG also reduced the MDI-induced ROS levels at the MCE stage, which may have affected adipogenesis.

During MCE, as the number of cells increases, proliferation-related signaling pathways are activated; representative pathways include the MAPK/ERK and PI3K/AKT pathways [21,35]. The MAPK/ERK and PI3K/AKT signaling pathways facilitate the transition of cells from the G_1_ phase to the S phase; therefore, the inhibition of these pathways suppresses the proliferation of 3T3-L1 cells. Citral, a major component of lemongrass oil, regulates adipogenic transcription factors by reducing PI3K/Akt signaling involved in cell survival, differentiation, and inflammatory responses [36]. Resveratrol, curcumin, (−)-epigallocatechin gallate, and cinnamyl alcohol [27,37,38,39] exhibit anti-obesity effects by inhibiting both the MAPK/ERK and PI3K/AKT signaling pathways and reducing the phosphorylation of ERK and AKT. IEG also inhibited adipogenesis-related factors by inhibiting the phosphorylation of ERK1/2 and AKT, which are related to cell proliferation.

## 5. Conclusions

We successfully demonstrated that IEG exerts an anti-adipogenic effect in 3T3-L1 cells by modulating MCE. At the MCE stage, IEG affects ROS generation, cell proliferation-related signaling pathways, including the MAPK/ERK and AKT signaling pathways, and cell cycle re-entry. Obesity causes numerous complications, emphasizing the importance of effective therapeutic strategies for obesity prevention. Naturally derived compounds can be a good alternative due to their low toxicity. Although this experiment was limited because it was conducted only in vitro, it is a meaningful result that shows the possibility of its use as an anti-obesity agent.

## Figures and Tables

**Table 1:** Primer sequences.

Gene		Primer Sequence (5′-3′)
*Pparg*	Forward	AAGGATTCATGACCAGGGAGTTCC
	Reverse	GCGGTCTCCACTGAGAATAATG
*Cebpα*	Forward	GTGGACAAGAACAGCAACGAGT
	Reverse	AGGCGGTCATTGTCACTGGTCAA
*Fapb4*	Forward	GTGGGCTTTGCCACAAGGAAAGT
	Reverse	GGTGATTTCATCGAATTCCACGCC
*Adipoq*	Forward	AGCCGCTTATGTGTATCGCTCAG
	Reverse	CCCGGAATGTTGCAGTAGAACT
*Fasn*	Forward	TGGGTTTGGTGAATTGTCTCCG
	Reverse	ACACGTTCATCACGAGGTCATG
*Cebpb*	Forward	AACAACATCGCGGTGCGCAA
	Reverse	AACAAGTTCCGCAGGGTGCTGA
*CyclinD1*	Forward	AAGCAGACCATCCGCAAGCA
	Reverse	GGTAGCAGGAGAGGAAGTTGTT
*Cdk6*	Forward	TTTTCAGATGGCCCTTACCTCG
	Reverse	CCACGAAAAAGAGGCTTTCTGC
*Cdk2*	Forward	CGAGCACCTGAAATTCTTCTGG
	Reverse	AGAGTCCGAAAGATCCGGAA
*CyclinB1*	Forward	GCATCTAAAGTCGGAGAGGT
	Reverse	GGTGTCCATTCACCGTTGTC
*18S*	Forward	GTAACCCGTTGAACCCCATT
	Reverse	CCATCCAATCGGTAGTAGCG

## Data Availability

All data supporting the findings of this study are available within this article.

## Supplementary Materials

The following are available online at https://www.mdpi.com/article/10.3390/nu16091262/s1, Figure S1: Protein expression of β-actin in 3T3-L1 cells at day 8; Figure S2: GAPDH mRNA expression level in adipocytes at 8 days; Figure S3: The 18S mRNA expression level in adipocytes at 8 days [40,41].
