# Supplementary material for: Isoeugenol Inhibits Adipogenesis in 3T3-L1 Preadipocytes with Impaired Mitotic Clonal Expansion
Source: Nutrients. 2024 Apr 24;16(9):1262. doi: 10.3390/nu16091262 (PMC11085592; doi:10.3390/nu16091262)
Supplement: Supplementary file 1 [file nutrients-16-01262-s001.zip › nutrients-2958626-supplementary.pdf]

Supplementary materials

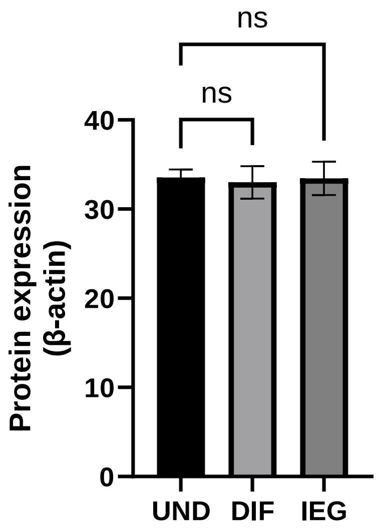

Figure S1. Stable protein expression of β-actin (reference gene) in 3T3-L1 preadipocytes and adipocytes at day 8 [41]. UND: preadipocytes, DIF: adipocytes, IEG: DIF+IEG treated adipocytes.

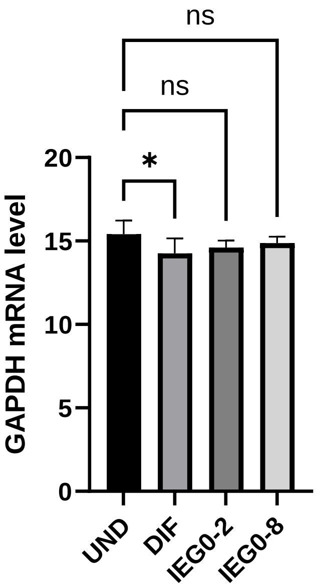

Figure S2. Decreased mRNA expression of GAPDH (reference gene) in 3T3-L1 adipocytes at 8 days [41]. UND: preadipocytes, DIF: adipocytes, IEG: DIF+IEG treated adipocytes.

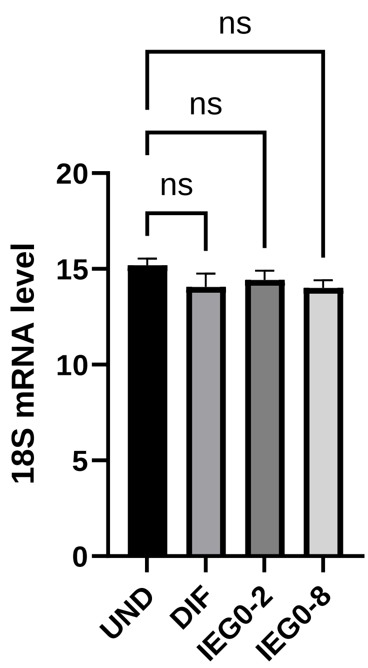

Figure S3. Stable mRNA expression of 18S (reference gene) in 3T3-L1 preadipocytes and adipocytes at 8 days [42]. UND: preadipocytes, DIF: adipocytes, IEG: DIF+IEG treated adipocytes.
